# Genetic predisposition to high BMI, ultra-processed food consumptions in childhood, and adiposity in young adulthood: a 17-year prospective cohort study of 3061 individuals

**DOI:** 10.1186/s12916-026-04764-5

**Published:** 2026-04-16

**Authors:** Ziyi Zhou, Qiaosen Chen, Zhi Li, Shi Zhang, Solange Parra-Soto, Anqi Wang, Mengxi Du, Xinwen Xu, Zhe Fang, Yujia Lu, Yu Chen Zhao, Rona J. Strawbridge, Patrick Ip, Naveed Satter, Nicholas J. Timpson, Mingyang Song, Carlos Celis-Morales, Marc J. Gunter, Jill P. Pell, Edward Giovannucci, Frederick K. Ho

**Affiliations:** 1 School of Health and Wellbeing, University of Glasgow, Glasgow, UK; 2 Department of Epidemiology, Harvard T.H. Chan School of Public Health, Boston, MA USA; 3 Division of Cardiology, Department of Medicine, Karolinska Institutet, Stockholm, Sweden; 4 Department of Nutrition, Harvard T.H. Chan School of Public Health, Boston, MA USA; 5 School of Cardiovascular and Metabolic Health, University of Glasgow, Glasgow, UK; 6 Department of Nutrition and Public Health, Universidad del Bío-Bío, Chillan, Chile; 7 Department of Metabolism, Digestion and Reproduction, Imperial College, London, UK; 8 Broad Institute of MIT and Harvard, Cambridge, MA USA; 9 Health Data Research UK, Glasgow, UK; 10 Department of Paediatrics and Adolescent Medicine, University of Hong Kong, Hong Kong, Hong Kong; 11 Department of Paediatrics and Adolescent Medicine, Hong Kong Children’s Hospital, Hong Kong, Hong Kong; 12 MRC Integrative Epidemiology Unit, University of Bristol, Bristol, UK; 13 Clinical and Translational Epidemiology Unit, Massachusetts General Hospitaland, Harvard Medical School , Boston, MA USA; 14 Division of Gastroenterology, Massachusetts General Hospitaland, Harvard Medical School , Boston, MA USA; 15 Human Performance Laboratory, Physical Activity and Health Research Unit, Universidad Catolica del Maule, EducationTalca, Chile; 16 High-Altitude Medicine Research Centre (CEIMA), Universidad Arturo Prat, Iquique, Chile; 17 Nutrition and Metabolism Branch, International Agency for Research on Cancer, World Health Organization, Lyon, France; 18 Department of Epidemiology and Biostatistics, School of Public Health, Imperial College London, London, UK

**Keywords:** ultra-processed food, adiposity, BMI, genetic susceptibility, obesity

## Abstract

**Background:**

Observational evidence suggests ultra-processed food (UPF) may contribute to obesity, but some people who consume a larger amount of UPF remain at normal weight. This study examined whether childhood UPF consumption was associated with obesity in early adulthood and whether the association was modified by genetic susceptibility to body mass index (BMI).

**Methods:**

This prospective cohort study included data from 3061 participants of the Avon Longitudinal Study of Parents and Children (ALSPAC) in England with follow-up from 7 to 24 years. UPF consumption was calculated from food diaries based on the NOVA classification. LDpred2 was used to construct a polygenic score (PGS) for body mass index (BMI). Linear regression models were used to estimate the association between UPF intake at 7 years and BMI at 24 years. The PGS-UPF interaction was examined to see whether genetic susceptibility modifies the association between childhood UPF consumption and early adulthood BMI.

**Results:**

Each 10% increase in the proportion of total energy intake coming from UPF at 7 was associated with 0.21 (95% CI 0.05–0.37) kg/m^2^ higher BMI at 24, after adjusting for BMI at 7, age, sex, ethnicity, physical activity, socioeconomic position, and total energy intake. There is evidence for PGS-BMI interaction (0.19; 95% CI 0.02–0.36), and the UPF-BMI association was only retained in children with the highest genetic predisposition to higher BMI (0.74, 95% CI 0.07–1.42) in the subgroup analysis.

**Conclusions:**

UPF consumption in childhood is only associated with early adulthood obesity among children more genetically predisposed to higher BMI.

**Supplementary Information:**

The online version contains supplementary material available at 10.1186/s12916-026-04764-5.

## Background

Ultra-processed foods (UPF), as defined by the NOVA food classification system, are industrially formulated products composed primarily, or entirely, of food-derived substances that have undergone extensive processing [1, 2]. These products include packaged snacks, carbonated soft drinks, instant noodles and ready-made meals. They are designed for convenience (ready-to-eat), extended shelf life (long-lasting), and highly palatability and, therefore, often incorporate additives and modified ingredients to enhance their taste, texture, and preservation [1, 3]. The consumption of UPFs and their components has been shown to be adversely associated with gut microbiota, systemic inflammation, insulin resistance, and body weight [2]. There is substantial variation in UPF consumption across countries and regions, but a global increase in their intake has been reported [1, 3].

Obesity is a growing public health concern globally. Several studies have suggested that higher consumption of UPFs may associated with greater BMI and weight gain, which in turn increase the risk of overweight and obesity [4, 5]. Most research on the relationship between UPF consumption and obesity has focused on middle-aged and older adults. A prospective study of 22,659 UK Biobank participants, aged 40–69 years, found that those with higher UPF consumption, identified using the NOVA classification, were 1.79 times more likely to develop obesity over 5 years of follow-up [6]. Studies on childhood UPF consumption primarily have been smaller and used cross-sectional designs. Therefore, understanding of the association between childhood UPF consumption and obesity is limited, despite the rising consumption of UPF in children [4, 7]. The US National Health and Nutrition Examination Survey reported that the total caloric intake derived from UPFs has risen from 61 to 67% over the past two decades among US children and adolescents aged 2–19 years [3, 8]. This rising trend is especially pronounced among individuals and families with lower socioeconomic status and educational levels, where obesity is more prevalent [7]. Importantly, the dietary patterns formed in childhood often persist into adulthood, likely resulting in long lasting health impact [7].

Whilst studies have shown an association between UPF consumption in childhood and obesity in young adulthood [9, 10], a sizable number of children with high UPF consumption do not develop obesity in childhood or later life. There may be factors that modify the association between UPF consumption and obesity. Among potential modifiers of the relationship between dietary risk factors and obesity, genetic predisposition to higher BMI is an obvious candidate, given its strong role in modifying the impact of other lifestyle factors in adulthood [11].

Higher overall diet quality appears to attenuate the effects of BMI-increasing genetic variants on adiposity, whereas greater intake of sugar-sweetened beverages strengthens these genetic effects [12]. However, evidence that UPF consumption specifically modifies the effects of genetic susceptibility to higher BMI remains limited, even though there are some potential plausible mechanisms [13], e.g. UPFs were shown to promote excess energy intake and rapid glycaemic and insulinaemic responses, which may be particularly relevant for individuals with variation in genes related to appetite or glucose regulation; and potential effects on gut microbiota and low-grade systemic inflammation [13]. Therefore, this study aims, firstly, to examine whether UPF consumption in childhood is associated with obesity in early adulthood, and, secondly, to determine whether the association between childhood UPF consumption and obesity differs according to genetic susceptibility to higher BMI.

## Methods

### Study design

The Avon Longitudinal Study of Parents and Children (ALSPAC) recruited 14,541 pregnant women residents in the Southwest of England, with an expected date of delivery between 1 st April 1991 and 31 st December 1992 [14]. The 15,645 offspring from these pregnancies and their parents have been followed up through structured questionnaires, measurements at clinical assessment visits and biological samples, as well as data linkage to routine health records [9, 14, 15]. Full details of the ALSPAC consent procedures are available on the study website (http://www.bristol.ac.uk/alspac/researchers/research-ethics/) [15].

This study included the 3,016 ALSPAC European offspring who parent-reported food diaries at 7 years of age and body mass index (BMI) measured at 24 years. The median (interquartile range) of follow-up was 17.0 (16.42–17.58) years. The participant selection flow chart, study design and timeline of data collection are shown in Additional File 1: Fig. S1 and S2.

### Exposures

#### Ultra-processed food consumption

Dietary data for children were collected using a 3-day food diary, which was given to parents before their child's clinic assessment at around 7 years of age (earliest time point) [16]. Caregivers recorded all food and beverage items consumed by the child over two weekdays and one weekend day (not necessarily consecutive) [16]. The recorded dietary data were reviewed by a nutritionist, coded using the Diet In, Data Out (DIDO) computer program, and linked to the 5th edition of the McCance and Widdowson British food composition tables for nutritional analysis [17].

To assess UPF intake, although the criteria vary across systems, the NOVA framework is among the most comprehensive and widely used [18]. Therefore, we applied the NOVA classification system, which categorises foods based on the extent and purpose of industrial processing [19]. Foods and beverages were classified into four groups: (1) unprocessed or minimally processed foods, such as fresh or frozen fruits, vegetables, milk, meat, and legumes; (2) processed culinary ingredients, including salt, sugar, and vegetable oils; (3) processed foods, made by adding salt, sugar, or other culinary ingredients to group 1 foods, such as canned vegetables, tinned fish, and freshly baked bread; and (4) UPFs, which are formulations of industrially processed ingredients, often containing additives like colours, flavours, or emulsifiers (Additional File 1: Table S1). The foods coded in ALSPAC is based on the UK National Diet and Nutrition Survey (NDNS), which included foods that could be classified in multiple NOVA categories (e.g. bread, yoghurt, and meat dishes) based on the level of industrial processing. We classify these foods based on the most consumed varieties from the NDNS 1997 (Additional File 1: Supplementary Methods). This year was chosen as it was the closet survey to the UPF measurement in ALSPAC (1998-1999) that included children/adolescent participants. To account for variations in total energy intake, UPF consumption was expressed as the percentage of total daily energy intake (kcal) derived from UPFs [20–22].

#### Genetic predisposition to BMI

Blood samples were collected from ALSPAC offspring at age 7 between 1998 and 2000. Samples were processed and stored by the Bristol Bioresource Laboratories (BBL) under an ISO9001-certified quality management system and Human Tissue Authority license.

All genotype data analysed in the present study were based on the Genome Reference Consortium Human Build 37 (GRCh37). The ALSPAC children were genotyped using the Illumina HumanHap550 quad genome-wide SNP genotyping platform by 23 and Me, with laboratory processing subcontracted to the Wellcome Trust Sanger Institute (Hinxton, UK) and the Laboratory Corporation of America (LapCorp; Burlington, NC, USA) [23]. Genotypes were subsequently imputed with MACH version 1.0.16, with CEPH individuals from phase 2 of the HapMap project used as the reference set [23].

Quality control procedures removed related individuals and individuals of non-European genetic ancestry. Full quality control information has been described on ALSPAC omics team (https://proposals.epi.bristol.ac.uk/alspac_omics_data_catalogue.html), and can also be found in Additional File 1: Supplementary Methods, respectively.

Quality control was performed at both the single nucleotide polymorphism (SNP) and individual level. At the SNP level, we excluded variants with a minor allele frequency (MAF) below 1%, an imputation information score less than 0.8, a minor allele count (MAC) of less than 10, and a Hardy–Weinberg Equilibrium (HWE) *p*-value lower than 1e − 8 to remove rare variants and potential genotyping errors. Additionally, we applied a genotype missingness threshold of 0.01, removing SNPs with more than 1% missing rate. At the individual level, we excluded participants with an individual missingness rate greater than 1% to ensure data integrity and minimise the impact of low-quality samples. A total of 6286 individuals and 660,451 SNPs passed these quality control filters. Detail of the SNPs quality control procedures and variant retention has been shown in Additional File 1: Fig. S3.

We performed principal component analysis (PCA) via the PLINK2 PCA command and obtained the top 10 genetic principal components (PCs) and those components were included in the linear regression to adjust for population stratification (Additional File 1: Table S2).

All the genome-wide association study (GWAS) were based on GRCh37 gene build. We extracted the GWAS summary data for BMI [24], from the previously published meta-analysis conducted by the GIANT consortium and UK Biobank, which included 456,426 participants, which did not include individuals from the ALSPAC cohort.

We constructed a polygenic score (PGS) for body mass index (BMI-PGS) using the LDpred2-auto algorithm, which applies Bayesian framework and accounts for linkage disequilibrium (LD, the correlation between genetic variants) to improve effect size estimation from GWAS data [23].

We used the LDpred2-auto model, which requires two key genetic architecture hyperparameters: (1) an estimate of SNP heritability (h2) and (2) proportion of causal variants (p) directly from the data and produced posterior SNP effect estimates by reweighting BMI GWAS summary statistics. The initial *h*^2^, prespecified by LD score regression, calibrates the strength of shrinkage for the effect sizes of genetic variants. The initial p, prespecified as a sequence from 1 − e4 to 0.2, reflects the levels of polygenicity. Starting from these values, LDpred2-auto alternates between updating LD-adjusted SNP effect estimates and re-estimating the architecture parameters from the data until convergence. After convergence, each individual’s BMI-PGS was calculated as the sum of LDpred2-auto–derived SNP weights multiplied by the corresponding genotype dosages (0/1/2) across all included harmonised variants. The coefficients of the developed BMI-PGS are shown in Additional File 2.

Based on summary statistics from a GWAS of BMI in individuals of European ancestry, a BMI polygenic score (BMI-PGS) was constructed using the LDpred2-auto algorithm. Analyses were restricted to high-quality variants overlapping with the HapMap 3 SNP set, as recommended for robust polygenic score construction. The BMI-PGS was standardized to *Z*-scores, and its distribution approximated a normal distribution centred at zero (Additional File 1: Fig. S4). There were 0.093 and 0.151 variance explained (R^2^) by the BMI-PGS for both BMI at age 7 and age 24 within the ALSPAC sample. The PGS was used as a continuous variable primarily, as well as a categorical variable based on the distribution of the data with a *z*-score between − 1 and 1 to categorical three levels: low (*z*-score < − 1), moderate (*z*-score between − 1 and 1), and high (*z*-score > 1) genetic predisposition. The two tail ends represent 16% of the population respectively. As a validation for external validity, the correlation of BMI-PGS with socioeconomic status (SES) and cardiovascular risk factors was calculated, which revealed good correlation (Additional File 1: Table S5). There were previous reports on associations of BMI-PGS with SES [25] and CVD risk factors [26].

### Outcomes

Adulthood BMI was measured, at around 24 years, in a clinic assessment following standardized procedures. Standing height, measured to the nearest centimetre, was recorded using a Harpenden wall-mounted stadiometer (Holtain Ltd, Crosswell, Crymych, UK) [9, 27–29]. Weight was measured to the nearest 0.1 kg using a Tanita TBF-401 (Model A, Tanita Corp., Tokyo, Japan) electronic body composition scale [27–29]. BMI was calculated as weight in kilograms divided by height in metres squared [9, 28]. Similarly, participants’ childhood BMI was ascertained in a clinic assessment at around 7 years and converted in age- and sex-specific *z*-scores to account for age-related variation in BMI during childhood and differences in growth trajectories by sex, including those related to pubertal timing.

### Covariates

The covariates selected were those that could affect the exposure and outcome variables, i.e. confounders. If the covariates were measured at multiple time points, we selected those that were immediately before the 7-year assessment, except for objectively measured physical activity. The following details were considered for each participant: ages at the clinic assessments during which baseline at age 7 and outcome at age 24 data were collected; sex (male or female) at age 7; ethnicity (white or other); objectively measured physical activity level (daily minutes of moderate and vigorous physical activity (MVPA)) at age 11; mean daily energy intake (a continuous variable) at age 7; BMI *z*-score at age 7, and socioeconomic status (quintile of the Index of Multiple Deprivation (IMD) 2000 at childbirth; maternal education level at age 32 weeks of gestation.

Physical activity was measured at approximately 11.8 years of age [30], and was the first objectively measured PA. Children wore accelerometers during waking hours, except when engaged in washing or doing water sports [30]. They were instructed to wear a uniaxial accelerometer (Model 7164; ActiGraph) for 7 consecutive days [28]. A validated method was used to derive time spent in MVPA, expressed in minutes/day in this study [30]. While this variable was measured after the UPF measurement, it is the most reliable measurement and acts as a proxy for PA at baseline.

Socioeconomic positions were measured using both area and individual level measures: The IMD is a widely used area-based measure of socioeconomic deprivation in England and is derived from aggregated data across seven domains of deprivation applied to postcode of residence [28]. Maternal education was collected from a self-reported questionnaire at 32 weeks of gestation and classified into three categories [22]: (i) low: Certificate of Secondary Education, Vocational or Ordinary- (O-) level, educational qualifications generally obtained at 17 years of age; (ii) intermediate: Advanced- (A-) level (subject specific qualifications generally obtained at age 18 years and required for university entry); (iii) high: university degree and above. An age- and sex-standardised BMI *z*-score was calculated for individuals at the age of 7 [31].

### Statistical analyses

The characteristics of participants stratified by quintile of baseline UPF consumption were summarised using means (standard deviations (SDs)) for numeric variables and frequencies (percentages) for categorical variables. Linear regression models were used to examine the association between UPF intake at 7 years of age and BMI outcomes at 24 years. PGS-UPF interactions were additionally assessed on a linear scale (linear regression), the interaction term in the model represents additive interaction. Further subgroup analyses were conducted to explore the association between UPF consumption and BMI across different levels of genetic risk (low, moderate, and high) using linear regression models.

All models were adjusted for baseline BMI z-score, age, sex, ethnicity, physical activity level, socioeconomic position, and total energy. The exposure variables were fitted on penalised cubic splines to investigate nonlinear associations between each UPFs consumption and the BMI in each imputed data. Non-linearity was tested by likelihood ratio tests. Missing covariate data were handled using multiple imputation of 10 imputed datasets. Analysis findings were pooled using Robin’s rule [32]. The study had 5.4% missing data overall and we selected 10, instead of 5 imputations to be more conservative [33]. The median and interquartile range of fraction of missing information were 10.4% (4.4–18.9%). The proportion of missing data has been shown in Additional File 1: Table S5.

## Results

A total of 3061 individuals were included after excluding those who did not complete any food diaries at 7 years and who have not had BMI measured at 24 (Additional File 1: Fig. S1 and S2). The included participants were slightly younger, more likely to be female, less deprived, and had a higher maternal education level (Additional File 1: Table S3). The majority (96.38%) of the individuals were of white ethnicity, to assess population structure in the genetic data, principal components (PCs) differed by parental ancestry (Additional File 1: Table S4), indicating that PCs captured ancestry-related population stratification. We therefore included the top 10 PCs as covariates in all genetic-adjusted regression models. Of these, 61% were female, and 21.74% had mothers with an undergraduate degree or higher.Those with higher childhood UPF consumptions were less likely to have mothers who had completed a degree and were likely to have higher adult BMI at age 17. The BMI PGS *z*-score was slightly lower in individuals with lower UPF consumption compared to those with higher UPF consumption. At follow-up, BMI z-scores were slightly higher in the higher UPF consumption groups compared to lower consumption group (Table 1).

**Table 1:** Characteristics by quartiles of childhood ultra-processed food consumption prior to multiple imputations

		**Quartile of baseline UPF consumption**				
	**Overall**	Q1 [39.41, 71.03)	Q2 [71.03, 77.20)	Q3 [77.20, 83.11)	Q4 [83.11, 100.00]	*P*
**Number**	3,061	766	765	765	765	
**Age, year, mean (SD)**						
Baseline (measurement of UPF)	7.50 (0.29)	7.50 (0.29)	7.49 (0.28)	7.50 (0.30)	7.50 (0.30)	0.944
Follow-up (measurement of BMI)	24.49 (0.80)	24.50 (0.79)	24.50 (0.80)	24.53 (0.79)	24.44 (0.81)	0.128
**Sex**						0.472
Female	1866 (61.00)	458 (59.87)	461 (60.34)	462 (60.39)	485 (63.40)	
Male	1193 (39.00)	307 (40.13)	303 (39.66)	303 (39.61)	280 (36.60)	
**Ethnicity**						0.688
Non-White	98 (3.62)	26 (3.93)	28 (4.06)	24 (3.55)	20 (2.94)	
White	2609 (96.38)	636 (96.07)	661 (95.94)	652 (96.45)	660 (97.06)	
**Index of multiple deprivation 2004, quintile, mean (SD)**	2.57 (1.46)	2.63 (1.48)	2.51 (1.47)	2.50 (1.45)	2.64 (1.44)	0.129
**Mother’s highest education**						< 0.001
CSE or lower	214 (7.42)	43 (5.97)	42 (5.78)	57 (8.02)	72 (9.92)	
Vocational	192 (6.66)	43 (7.29)	48 (6.60)	46 (6.47)	55 (7.58)	
O level	973 (33.74)	208 (28.89)	240 (33.01)	247 (34.47)	278 (38.29)	
A level	878 (30.44)	235 (32.64)	229 (31.50)	214 (30.10)	200 (27.55)	
Degree	627 (21.74)	191 (26.53)	168 (23.11)	147 (20.68)	121 (16.67)	
**MVPA, min/day, mean (SD)**	21.68 (14.22)	22.20 (14.30)	21.70 (14.18)	21.46 (14.27)	21.56 (14.14)	0.916
**BMI PGS *z*-score, mean (SD)**	− 0.08 (1.01)	− 0.09 (1.04)	− 0.11 (1.02)	− 0.07 (1.01)	− 0.05 (0.97)	0.817
Lower (< − 1)	372 (18.09)	101 (19.73)	97 (18.65)	91 (18.09)	83 (15.93)	
Moderate [− 1, 1]	1,405 (68.34)	336 (65.63)	352 (67.69)	345 (68.59)	372 (71.40)	
Higher (> 1)	279 (13.09)	75 (14.65)	71 (13.65)	67 (13.32)	66 (12.67)	
**%E from UPF at baseline, %E, mean (SD)**	77 (9)	65 (6)	74 (2)	80 (2)	88 (4)	< 0.001
**Total energy intake at baseline, mean (SD)**	1698.71 (302.49)	1699.35 (311.58)	1704.60 (299.44)	1704.16 (307.26)	1686.72 (291.52)	0.627
**BMI *z*-score in childhood, mean (SD)**	0.10 (1.02)	0.11 (1.01)	0.10 (0.98)	0.12 (1.02)	0.07 (1.06)	0.806
**BMI in adulthood (17 years of age), mean (SD)**	24.81 (5.04)	24.53 (4.51)	24.48 (4.69)	25.30 (5.60)	24.93 (5.24)	0.004

*CSE *Certificate of Secondary Education, *MVPA *moderate-to-vigorous intensity physical activity, *UPF *ultra-processed food; *%E*, % of total energy intake

Unless otherwise indicated, data are expressed as No. (%) of children. Percentages are rounded and may not add up to 100%

Table 2 shows the association between childhood UPF consumption and obesity in adulthood. Childhood UPF consumption was associated with adulthood BMI independent of potential confounders. When adjusted for age, sex, ethnicity, mother’s highest education, IMD quintile, baseline BMI *z*-score, total energy intake, physical activity level, BMI-PGS, and top 10 genetic principal components, a 10% higher energy intake from UPF was associated with a 0.21 unit (95% CI 0.05–0.37) higher BMI. This was equivalent to 0.79% and 0.05-SD of the mean values in this population. There was no evidence that the association between UPF and BMI was nonlinear (Additional File 1: Fig. S5).

**Table 2:** Associations between childhood UPF consumption (per 10% E) and adulthood BMI

	***β* coefficient (95%CI)**	***P*-value**
Model 1	0.21 (0.05–0.37)	0.011
Model 2	0.21 (0.05–0.37)	0.011
Model 3	0.21 (0.05–0.37)	0.009

Model 1 adjusted for age, sex, ethnicity, mother’s highest education, deprivation and baseline BMI z-scores; Model 2 additionally adjusted for total energy intake and moderate to vigorous physical activity; Model 3 additionally adjusted for corresponding PGS and top 10 genetic principal components

UPF consumption per 10% E: per 10% of total energy intake that comes from UPFs

**Fig. 1:**
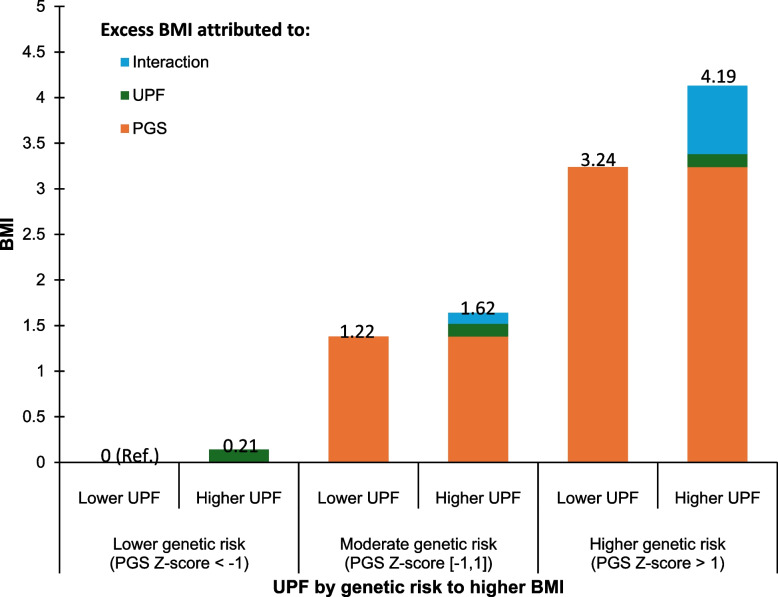
Additive interaction of UPF at age 7 and adulthood (age 24) adiposity in BMI-PRS subgroups. Adjusted for age, sex, ethnicity, mother’s highest education, deprivation and baseline BMI z-scores, total energy intake, and physical activity. UPF was a continuous variable, and ‘Higher UPF’ indicates 10% higher % energy intake from UPF

Table 3 shows the interaction between PGS and UPF on BMI. Evidence for an interaction was observed between BMI-PGS and UPF consumption in relation to adulthood BMI (*p* = 0.032). Figure 1 illustrates the additive interaction of UPF consumption and PGS on BMI. Compared with people with lower UPF consumption and lower PGS (*z*-score < − 1), those with higher UPF consumption and higher PGS (*z*-score > 1) were at disproportionately higher BMI (4.19 kg/m^2^), compared with those who only had higher PGS (3.24 kg/m^2^) and those who only had higher UPF (0.21 kg/m^2^). In the subgroup analyses (Table 4), the association between childhood UPF consumption and BMI in adulthood was apparently stronger in individuals with higher genetic predisposition to higher BMI (coefficient 0.74, 95% CI: 0.07–1.42).

**Table 3:** Interaction between childhood UPF consumption and continuous BMI-PGS on early adulthood BMI

Outcome	Model term	*β* coefficient (95%CI)	*P*-value
BMI	UPF	0.21 (0.05–0.37)	0.011
BMI	BMI PGS	1.22 (1.04–1.41)	< 0.001
BMI	UPF x BMI PGS	0.19 (0.02–0.36)	0.032

Adjusted for age, sex, ethnicity, mother’s highest education, deprivation, baseline BMI *z*-scores, total energy intake, and physical activity

Both UPF (per 10% energy intake) and BMI-PGS (*z*-score) were continuous variables

**Table 4:** Subgroup analyses of UPF at age 7 and adulthood adiposity in BMI-PRS subgroups

**Subgroup**	Number in subgroup	*β* coefficient (95%CI)	*P*-value
Low genetic risk	472	0.09 (− 0.19–0.36)	0.549
Moderate genetic risk	2,246	0.14 (− 0.04–0.32)	0.120
Higher genetic risk	343	0.74 (0.07–1.42)	0.031

Adjusted age at 7 and 24 years, sex, ethnicity, mother’s highest education, deprivation, baseline BMI *z*-scores, total energy intake, and moderate to vigorous physical activity

## Discussion

### Principal findings

In this population cohort study, we observed a linear association between UPF consumption in early childhood and young adulthood BMI after adjustment for potential confounders. More importantly, the hypothesis was confirmed as in subgroup analysis, the association between early childhood UPF and young adulthood BMI was specific to individuals with a high genetic predisposition to higher BMI, with a formal interaction between genetic propensity towards obesity and UPF intake for young adulthood BMI.

### Comparison with existing studies

Previous studies have focused on UPF consumption in adulthood [5], especially in middle age and old age [34], or have used cross-sectional designs to explore the association between childhood UPF consumption and adiposity in childhood, adolescence or early adulthood [4, 35]. For example, a Brazilian cross-sectional study of 30,243 individuals aged 10–39 years found no significant association between higher UPF consumption and BMI, obesity, or excess weight in adolescence and young adulthood [36].

Cohort studies of UPF consumption in childhood and obesity in adulthood have been small in number. A small prospective cohort study of 307 children aged 4 years showed similar findings to the present study with a 0.7 cm increase in waist circumference at 8 years of age for every 10% increase in UPF energy intake [37]. A previous study of 9025 children in ALSPAC demonstrated that UPF consumption at 7 years of age was associated with growth trajectories up to the age of 24 years [9]; BMI trajectory increased by an additional 0.06 (95% CI, 0.04–0.08) units per year in the highest UPF consumption group compared with the lowest. Similarly, waist circumference trajectory increased by an 0.17 (95% CI, 0.11–0.22) cm per year. The present study builds on these findings by demonstrating a significant interaction with genetic predisposition, whereby the association between childhood UPF consumption and obesity in adulthood was only significant among individuals with a genetic predisposition to higher BMI. This important finding shows that people susceptible to overeating from a genetic perspective will do so when more apparently tasty foods (UPF) are marketed and sold widely.

### Mechanisms

UPFs have been postulated to be associated with excess energy intake and weight gain because they are less satiating and elicit greater glycaemic response compared to minimally processed foods [38, 39]. Both mechanisms could be used to explain this study’s results. Even though total calorie intake at 7 was adjusted, imprecision measurement could mean residual association via excess eating because of UPF. It is also possible that UPF at 7 could lead to excess eating in later childhood. On the other hand, if there is a link beyond total calorie intake, several potential mechanisms might link UPF consumption to metabolic dysfunction, including impaired insulin regulation, increased nutrient storage in adipose tissue, and reduction in postprandial energy expenditure by 50%, exacerbating potential metabolic inefficiencies [34, 40–42]. There are other hypotheses on why UPF could lead to obesity, such as reduced fibre intake [43], leading to altered gut microbiota [44, 45], and in turn impaired energy metabolism [46]. Fiber intake is the one food substance with plentiful evidence, including randomised trials, for an association with.

lower weight when taken in greater amounts, so if fibre intake is lowered over time with more UPF intake, this would potentiate weight gain. While childhood and adolescence are characterised by high metabolic activity and energy expenditure, which may temporarily offset UPF-related obesity, the long-term consequences of habitual UPF consumption may only become apparent until young adulthood as we showed in this study. This underscores the need for early nutritional interventions to mitigate lifelong metabolic risks [9].

Although our BMI-PGS is genome-wide and not limited to a small set of loci, BMI GWAS signals are consistently enriched for CNS/appetite-related biology (e.g. FTO/MC4R and other neuronal loci), supporting a plausible mechanism whereby UPF exposure magnifies genetically influenced differences in appetite regulation and eating behaviour.

### Strengths and limitations of this study

Few prospective studies have examined UPF intake during childhood and adopted a longitudinal approach, following participants from childhood into young adulthood. This study explored a prospective study of the association between childhood UPF consumption and young adulthood BMI. The data also show that there was a linear dose-dependent relationship between UPF consumption and adulthood BMI. Most importantly, the novel finding that genetic predisposition to higher BMI modifies the relationship points to show important gene-environment interactions, which may ultimately help targeting of interventions through precision public health.

However, several limitations should be acknowledged. Firstly, potential misclassification of diet may arise from the limitations of the NOVA classification system and the calculation of nutrient intakes. A key concern is the conceptual heterogeneity within the NOVA system: individuals can achieve similar levels of UPF consumption while consuming very different types of foods, potentially with distinct health implications. In addition, there was no detailed processing information of the foods included in ALSPAC, some foods (e.g. bread, yoghurt, meat and dishes) were classified based on the majority varieties being consumed according to the UK NDNS (Additional File 1: Supplementary Methods). This inevitably introduced inaccuracy in the classification. Nevertheless, the risk of differential misclassification is likely minimised in our study, given the detailed food descriptions provided through food diaries in the ALSPAC cohort, and any residual inaccuracies are unlikely to be systematic. It should also be noted that UPF consumption at 7 years in this study is likely to reflect chronic consumption of UPF. We only included consumption data at age 7 since it was the only time point with parent-reported UPF, and there were no comparable measurements at a different time point. The lack of repeated measures could lead to non-differential misclassification in the form of regression dilution bias, resulting in underestimation. Secondly, even though we interpreted the statistical interaction between PGS and UPF to be PGS modifying UPF’s effect, it is also possible that UPF consumption modifies genetic effects and it is not possible to distinguish from this design. Thirdly, PA was measured after UPF measurement and thus the findings could be subject to overadjustment bias. However, even if this occurs our results still provide a conservative estimate. Fourthly, as with any observational studies, our findings cannot establish causality due to residual confounding. One notable confounder is parental BMI or BMI-PGS, which could be a cause for offspring’s UPF consumption as well as BMI. Lastly, ALSPAC is based on a region in England, with predominantly white people, limiting the generalisability of our findings in other populations. The inclusion criteria of this study likely amplified the selection bias especially in over-representing people of higher socioeconomic status. However, socioeconomic status does not appear to be a collider and thus the findings should still be internally valid.

## Conclusions

UPF consumption in childhood is independently associated with early adulthood obesity but appears to be so much more so among individuals with a high genetic predisposition to higher BMI. Larger studies using the same approach would help to validate these findings.

## Supplementary Information

Additional file 1. Supplementary methods. Supplementary Figure 1. Participants flowchart. Supplementary Figure 2. Study design and timeline of data collection. Supplementary Figure 3. Flowchart of SNP quality controlsteps and SNP retention. Supplementary Figure 4. Distribution of polygenic score for BMI. Supplementary Figure 5. Non-linear association between childhood UPF consumption and adulthood adiposity in 10 imputed data. Supplementary Table 1. The NOVA food classification system and its food. Supplementary Table 2. The proportion of variance explained by the top 10 PCs from the PCA. Supplementary Table 3. Participant characteristics by inclusion and number of missing data. Supplementary Table 4. Association between PCA components and parental ethnicity. Supplementary Table 5. Association between SES, CVD risk factor with BMI-PGS.

Additional file 2. Coefficients of the BMI-PGS.

## Data Availability

Consent for publication not applicable. This manuscript does not contain any individual person’s identifiable data, images, or videos. The data used in this study were obtained from the Avon Longitudinal Study of Parents and Children under application number B3876. ALSPAC data are available through a managed access process. Information on the data access process is available via the ALSPAC data access webpages (https://www.bristol.ac.uk/media-library/sites/alspac/documents/researchers/data-access/ALSPAC_Access_Policy.pdf) [47]. Due to participant confidentiality and ethical governance, individual-level ALSPAC data are not posted publicly, but can be accessed by eligible researchers through the formal application procedure.

## Abbreviations

UPF: Ultra-processed food
BMI: Body mass index
PGS: Polygenic scores
ALSPAC: Avon Longitudinal Study of Parents and Children
DIDO: Diet In, Data Out
BBL: Bristol Bioresource Laboratories
GRCh37: Consortium Human Build 37
SNP: Single nucleotide polymorphism
MAC: Minor allele count
HWE: Hardy-Weinberg Equilibrium
PCA: Principal component analysis
PCs: Principal components
GWAS: Genome-wide association study
BMI-PGS: Polygenic score for body mass index
LD: Linkage disequilibrium
SES: Socioeconomic status
SDs: Standard deviations
