# Supplementary material for: Genetic predisposition to high BMI, ultra-processed food consumptions in childhood, and adiposity in young adulthood: a 17-year prospective cohort study of 3061 individuals
Source: BMC Med. 2026 Apr 16;24:251. doi: 10.1186/s12916-026-04764-5 (PMC13088704; doi:10.1186/s12916-026-04764-5)
Supplement: Supplementary file 1 — Additional file 1. Supplementary methods. Supplementary Figure 1. Participants flowchart. Supplementary Figure 2. Study design and timeline of data collection. Supplementary Figure 3. Flowchart of SNP quality controlsteps and SNP retention. Supplementary Figure 4. Distribution of polygenic score for BMI. Supplementary Figure 5. Non-linear association between childhood UPF consumption and adulthood adiposity in 10 imputed data. Supplementary Table 1. The NOVA food classification system and its food. Supplementary Table 2. The proportion of variance explained by the top 10 PCs from the PCA. Supplementary Table 3. Participant characteristics by inclusion and number of missing data. Supplementary Table 4. Association between PCA components and parental ethnicity. Supplementary Table 5. Association between SES, CVD risk factor with BMI-PGS. [file 12916_2026_4764_MOESM1_ESM.docx]

**Additional File 1**

**Supplementary Methods**

**GWAS data generation and quality control**

ALSPAC children were genotyped using the Illumina HumanHap550 quad chip genotyping platforms. The resulting raw genome-wide data were subjected to standard quality control methods. Individuals were excluded on the basis of gender mismatches; minimal or excessive heterozygosity; disproportionate levels of individual missingness (>3%) and insufficient sample replication (IBD < 0.8). Population stratification was assessed by multidimensional scaling analysis and compared with Hapmap II (release 22) European descent (CEU), Han Chinese, Japanese and Yoruba reference populations; all individuals with non-European ancestry were removed. Related subjects that passed all other quality control thresholds were retained during subsequent phasing and imputation.

This gave 8,237 eligible children with available genotype data after exclusion of related subjects using cryptic relatedness measures described previously.

**Determining NOVA categories in ambiguous foods**

Food consumption in ALSPAC was coded for aggregated food groups in UK National Diet and Nutrition Survey (NSNS) as shown in Supplementary Table 1. Some foods could be classified into multiple groups in the NOVA classification system, depending on the level of industrial processing and/or additives added. To classify these items, we examined the individual-level consumption data in the NDNS 1997 (n=1,701; aged 4-18 years) (1). NOVA classification was then determined based on the majority (>50% by weight) of the consumed variety. For example, less than 1% of the white bread consumed were reported to be home made and majority of the varieties were commercial brands which likely to have emulsifiers and other additives added. Therefore, white bread in this study was classified as group 4 (ultra-processed food) even though homemade or freshly baked bread are group 3 (processed food). Similarly, yoghurt could be group 1 (plain / natural) or group 4 (with sugar / flavourings added). The data from NDNS 1997 showed that majority (90%) of the yoghurt consumed were flavoured (mostly fruit or chocolate flavourings), and, therefore, yoghurt was classified as group 4 (ultra-processed food). In contrast, even though ‘chicken, turkey, and dishes’ included both read-to-heat dishes (e.g. ‘chilli con carne with turkey mince, mushrooms & kidney beans’) that is group 4, majority of the consumed were from group 3 (e.g. roast chicken, turkey ham). Therefore, ‘chicken, turkey, and dishes’ was classified as group 3.

**Reference**

1. Office for National Statistics. Social Survey Division, Medical Research Council. Resource Centre for Human Nutrition Research, Ministry of Agriculture, Fisheries and Food, Department of Health. (2001). National Diet and Nutrition Survey : Young People Aged 4 to 18 Years, 1997. [data collection]. UK Data Service. SN: 4243, DOI: http://doi.org/10.5255/UKDA-SN-4243-1

**Supplementary Figure 1. Participants flowchart**

15,645 individuals enrolled in ALSPAC

7,268 individuals

8,377 did not have food diaries completed at 7 years

4,207 have not had BMI measured at 24 years

3,061 individuals

included in analysis

**Supplementary Figure 2. Study design and timeline of data collection.**

**
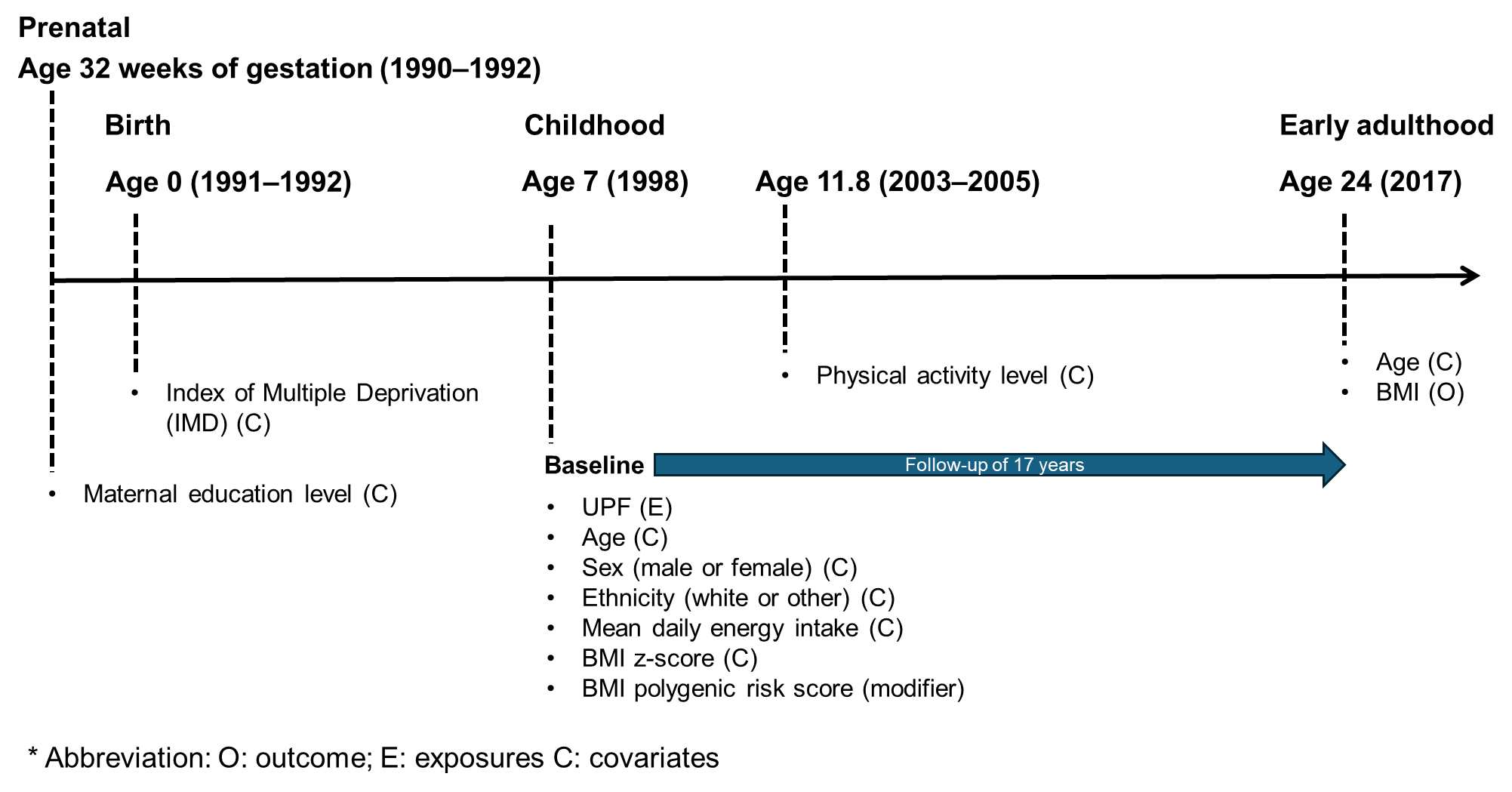
**

**Supplementary Figure 3. Flowchart of SNP quality control (QC) steps and SNP retention.**

**Initial GWAS**

SNP Count: 2,336,065

SNPs Lost: 0

Percent Lost: 0

Retention: 100%

**After QC merge**

SNP Count: 1,640,858

SNPs Lost: 695,207

Percent Lost: 29.76%

Retention: 70.24%

**After freq1/info filter**

SNP Count: 1,543,723

SNPs Lost: 97,135

Percent Lost: 5.92%

Retention: 66.08%

**After Map HapMap 3plus filter**

SNP Count: 661,493

SNPs Lost: 882,230

Percent Lost: 57.15%

Retention: 28.32%

**After SNP match**

SNP Count: 661,489

SNPs Lost: 4

Percent Lost: 0

Retention: 28.32%

**After MAF filter**

SNP Count: 660,451

SNPs Lost: 1,038

Percent Lost: 0.16%

Retention: 28.27%

**Supplementary Figure 4. Distribution of polygenic score for BMI**

**
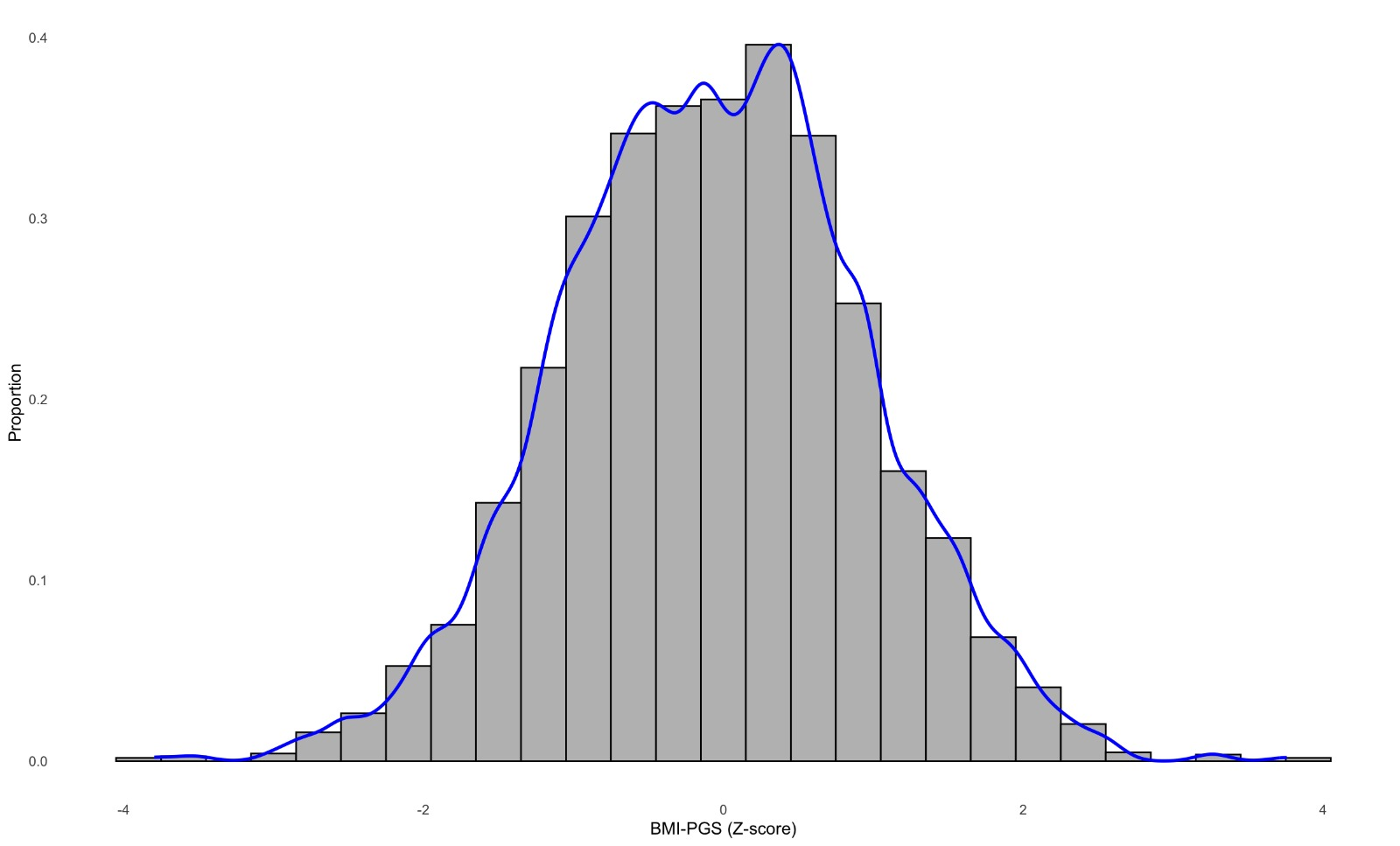
**

**Supplementary Figure 5.** **Non-linear association between childhood UPF consumption and adulthood adiposity in 10 imputed data**

Adjusted age at 7 and 24 years, sex, ethnicity, mother’s highest education, deprivation, baseline BMI z-score, total energy intake, moderate to vigorous physical activity and top 10 genetic principal components.

**Supplementary Table 1. The NOVA food classification system and its food**

| **NOVA Group** | **Definition** | **Classification in ALSPAC** |
| --- | --- | --- |
| **Group 1**  Unprocessed or minimally processed foods | *Unprocessed*  Edible parts of plants (fruit, seeds, leaves, stems, roots, tubers) or of or from animals (muscle, fat, offal, eggs, milk), and also fungi, algae, all after separation from nature. Spring and tap water.  *Minimally processed*  Unprocessed foods altered by industrial processes such as removal of inedible or unwanted parts, drying, powdering, squeezing, crushing, grinding, fractioning, steaming, poaching, boiling, roasting, and pasteurization, chilling, freezing, placing in containers, vacuum packaging, non-alcoholic fermentation, and other methods that do not add salt, sugar, oils or fats or other food substances to the original food.  The main aim of these processes is to extend the life of unprocessed foods, enabling their storage for longer use, or to make them edible, and, often, to make their preparation easier or more diverse.  Infrequently, minimally processed foods contain  additives that prolong product duration, protect  original properties or prevent proliferation of  microorganisms. | Raw carrots; cooked carrots; green leafy vegetables; peas; green and runner beans; cooked and canned tomatoes; raw tomatoes; other salad and raw vegetables; other cooked vegetables; legumes; whole milk; semi-skimmed milk; skimmed milk; soya milk; goat and sheep milk; other milk and cream; citrus fruit; apples and pears; bananas; other fruit; nuts; water and flavoured water; herbs and spices |
| **Group 2**  Processed culinary ingredients | Substances obtained directly from group 1 foods or from nature by industrial processes such as pressing, centrifuging, refining, extracting or mining.  Used to prepare, season and cook group 1 foods. May contain additives that prolong product duration, protect original properties or prevent proliferation of microorganisms. | Butter; polyunsaturated cooking fat; non-polyunsaturated cooking fat; salty flavourings; herbal tea; tea infusion |
| **Group 3**  Processed foods | Products made by adding salt, oil, sugar or other group 2 ingredients to group 1 foods, using preservation methods such as canning and bottling, and, in the case of breads and cheeses, using nonalcoholic fermentation.  Processes and ingredients here are designed to increase the durability of group 1 foods and make them more enjoyable by modifying or enhancing their sensory qualities. They may contain additives that prolong product duration, protect original properties, or prevent proliferation of microorganisms. | Oily fish; other white fish, shellfish, fish dishes; offal (excluding liver); eggs and egg dishes; coated and fried white fish, shellfish; coated chicken and turkey; chicken, turkey and dishes; liver and dishes; lamb and dishes; pork and dishes; beef and dishes; ham and bacon; other potatoes; cheese; fruit canned in juice |
| **Group 4**  Ultra-processed foods | Formulations of ingredients, mostly of exclusive industrial use, made by a series of industrial processes, many requiring sophisticated equipment and technology (hence ‘ultra-processed’). Processes used to make ultra-processed foods include the fractioning of whole foods into substances, chemical modifications of these substances, assembly of unmodified and modified food substances using industrial techniques such as extrusion, moulding and pre-frying; use of additives at various stages of manufacture whose functions include making the final product palatable or hyper-palatable; and sophisticated packaging, usually with plastic and other synthetic materials. Ingredients include sugar, oils or fats, or salt, generally in combination, and substances that are sources of energy and nutrients that are of no or rare culinary use such as high fructose corn syrup, hydrogenated or interesterified oils, and protein isolates; classes of additives whose function is to make the final product palatable or more appealing such as flavours, flavour enhancers, colours, emulsifiers, and sweeteners, thickeners, and anti-foaming, bulking, carbonating, foaming, gelling, and glazing agents; and additives that prolong product duration, protect original properties or prevent proliferation of microorganisms.  Processes and ingredients used to manufacture  ultra-processed foods are designed to create highly  profitable products (low-cost ingredients, long shelf life, emphatic branding), convenient (ready-to-consume) hyper-palatable products liable to  displace freshly prepared dishes and meals made  from all other NOVA food groups. | Breakfast cereals; other breakfast cereals; sweet biscuits; yoghurt and fromage frais; puddings and ice creams; buns, cakes, pastries and fruit pie; crisps and savoury snacks; sugar confectionery; chocolate confectionery; sugar, preserves and sweet spreads; baked beans; meat pies and pastries; burgers and kebabs; sausages; other meat and meat products; white bread; brown and granary bread; soft grain white bread; whole meal bread; other bread; full-fat polyunsaturated margarine; low-fat polyunsaturated margarine; full-fat non-polyunsaturated margarine; low-fat non-polyunsaturated margarine; fried/roast potatoes and chips; vegetable dishes; fruit juice; fruit canned in syrup; pasta, rice, pizza etc; soup; normal squashes and cordials; normal fizzy drinks and made-up squash; diet squashes and cordials; diet fizzy drinks and made-up squash;  alcoholic drinks; milk-based sauces; tomato-based sauces; other sauces; sugar-free confectionery; savoury biscuits and crackers; powdered drinks e.g. drinking chocolate; soya products; instant coffee granules/powder; coffee infusion/made-up instant coffee |

**Supplementary Table 2.** **The proportion of variance explained by the top 10 PCs from the PCA.**

| **Principal component** | **Proportion of variance (%)** |
| --- | --- |
| PC1 | 7.933 |
| PC2 | 5.122 |
| PC3 | 4.712 |
| PC4 | 4.476 |
| PC5 | 3.740 |
| PC6 | 3.490 |
| PC7 | 3.313 |
| PC8 | 3.263 |
| PC9 | 3.149 |
| PC10 | 3.119 |

**Supplementary Table 3. Participant characteristics by inclusion and number of missing data.**

|  | **Excluded** | **Included** | **Standardised Mean Difference** | | **95% CI** |
| --- | --- | --- | --- | --- | --- |
| **Number** | 12,584 | 3,061 |  |  | |
| **Age, year, mean (SD)** |  |  |  |  | |
| Baseline (measurement of UPF) | 7.56 (0.33) | 7.50 (0.29) | 0.20 | 0.15, 0.24 | |
| Missing | 7,370 | 0 |  |  | |
| Follow-up (measurement of BMI) | 24.45 (0.87) | 24.49 (0.80) | -0.05 | -0.12, 0.03 | |
| Missing | 11,627 | 0 |  |  | |
| **Sex** |  |  | 0.31 | 0.27, 0.35 | |
| Female | 5,480 (45.78%) | 1,866 (61%) |  |  | |
| Male | 6,491 (54.22%) | 1,193 (39%) |  |  | |
| Missing | 613 | 2 |  |  | |
| **Non-white ethnicity** | 186 (3.61%) | 98 (3.62%) | 0.00 | -0.05, 0.05 | |
| Missing | 7,436 | 354 |  |  | |
| **Index of multiple deprivation 2004, quintile** |  |  | 0.37 | 0.33, 0.41 | |
| 1 – Least deprived | 2,238 (22.31%) | 942 (34.91%) |  |  | |
| 2 | 1,565 (15.60%) | 483 (17.90%) |  |  | |
| 3 | 1,704 (16.98%) | 466 (17.27%) |  |  | |
| 4 | 1,810 (18.04%) | 407 (15.09%) |  |  | |
| 5 – Most deprived | 2,716 (27.07%) | 400 (14.83%) |  |  | |
| Missing | 2,551 | 363 |  |  | |
| **Mother’s highest education** |  |  | -0.59 | -0.63, -0.55 | |
| CSE or lower | 2,310 (24.10%) | 214 (7.42%) |  |  | |
| Vocational | 1,037 (10.82%) | 192 (6.66%) |  |  | |
| O level | 3,346 (34.91%) | 973 (33.74%) |  |  | |
| A level | 1,914 (19.97%) | 878 (30.44%) |  |  | |
| Degree | 979 (10.21%) | 627 (21.74%) |  |  | |
| Missing | 2,998 | 177 |  |  | |
| **MVPA, min/day, mean (SD)** | 24.09 (15.97) | 21.68 (14.22) | 0.16 | 0.11, 0.21 | |
| Missing | 9,324 | 734 |  |  | |
| **BMI PGS z-score, mean (SD)** | 0.04 (0.99) | -0.08 (1.01) | 0.12 | 0.06, 0.17 | |
| Lower (< -1) | 608 (14.64%) | 372 (18.09%) |  |  | |
| Moderate [-1, 1] | 2,847 (68.55%) | 1,405 (68.34%) |  |  | |
| Higher (> 1) | 698 (16.81%) | 279 (13.57%) |  |  | |
| Missing | 8,431 | 1,005 |  |  | |
| **%E from UPF at baseline, %E, mean (SD)** | 77.16 (9.34) | 76.61 (9.19) | 0.06 | 0.01, 0.11 | |
| Missing | 8,377 | 0 |  |  | |
| **Total energy intake at baseline, mean (SD)** | 1,705.33 (322.30) | 1,698.71 (302.49) | 0.02 | -0.03, 0.07 | |
| Missing | 8,374 | 0 |  |  | |
| **BMI z-score in childhood, mean (SD)** | 0.16 (1.06) | 0.10 (1.02) | 0.06 | 0.01, 0.10 | |
| Missing | 7,435 | 14 |  |  | |
| **BMI in adulthood (17 years of age), mean (SD)** | 25.30 (5.19) | 24.81 (5.04) | 0.10 | 0.02, 0.17 | |
| Missing | 11,671 | 0 |  |  | |

Abbreviations: CSE, Certificate of Secondary Education; MVPA, moderate-to-vigorous intensity physical activity; UPF, ultra processed food; %E, % of total energy intake.

Unless otherwise indicated, data are expressed as No. (%) of children. Percentages are rounded and may not add up to 100%.

**Supplementary Table 4.** **Association between PCA components and parental ethnicity.**

| **Principal component** | **Comparison** | **Beta** | **P-value** |
| --- | --- | --- | --- |
| PC1 | Father white only | -0.0036 | 1.53E-01 |
| PC1 | Mother white only | -0.0009 | 6.67E-01 |
| **PC1** | **Neither parent white** | **0.008** | **6.07E-03** |
| PC2 | Father white only | -0.0011 | 6.61E-01 |
| PC2 | Mother white only | 0.0001 | 9.72E-01 |
| **PC2** | **Neither parent white** | **0.0065** | **2.47E-02** |
| PC3 | Father white only | -0.0003 | 9.02E-01 |
| PC3 | Mother white only | 0.0009 | 6.70E-01 |
| PC3 | Neither parent white | 0.0054 | 6.08E-02 |
| PC4 | Father white only | -0.0021 | 3.86E-01 |
| PC4 | Mother white only | 0.003 | 1.40E-01 |
| PC4 | Neither parent white | -0.0017 | 5.59E-01 |
| PC5 | Father white only | -0.0013 | 6.02E-01 |
| PC5 | Mother white only | 0.0019 | 3.66E-01 |
| PC5 | Neither parent white | 0.0051 | 7.83E-02 |
| PC6 | Father white only | 0.0013 | 5.87E-01 |
| PC6 | Mother white only | 0.0012 | 5.48E-01 |
| PC6 | Neither parent white | -0.0013 | 6.52E-01 |
| PC7 | Father white only | 0.0017 | 5.06E-01 |
| PC7 | Mother white only | 0.0017 | 4.10E-01 |
| PC7 | Neither parent white | 0.0019 | 5.21E-01 |
| PC8 | Father white only | -0.0006 | 8.06E-01 |
| PC8 | Mother white only | -0.0026 | 2.05E-01 |
| PC8 | Neither parent white | 0.0017 | 5.61E-01 |
| PC9 | Father white only | 0.0032 | 2.02E-01 |
| PC9 | Mother white only | 0.0017 | 4.14E-01 |
| PC9 | Neither parent white | 0.0015 | 5.94E-01 |
| PC10 | Father white only | -0.0024 | 3.28E-01 |
| PC10 | Mother white only | -0.0022 | 2.82E-01 |
| PC10 | Neither parent white | 0.001 | 7.18E-01 |

Reference group is 'both_1' (both parents coded as white). Parental ethnicity variables use '1' to indicate white ancestry; non-1 values correspond to other ancestries. Rows highlighted in gold denote nominal significance (p < 0.05).

**Supplementary Table 5. Association between SES, CVD risk factor with BMI-PGS.**

|  | Coefficient (95% CI) | P-value |
| --- | --- | --- |
| Mother’s highest education | -0.12 (-0.16 - -0.09) | <0.001 |
| Index of multiple deprivation 2004, quintile | 0.05 (0.02 - 0.08) | <0.001 |
| Diastolic blood pressure | 0.01 (0.04 - 0.01) | <0.001 |
| Systolic blood pressure | 0.01 (0.01 - 0.02) | <0.001 |
| LDL cholesterol | 0.11 (0.05 - 0.17) | <0.001 |
| Insulin | 0.02 (0.01 - 0.02) | <0.001 |
| Fasting glucose | 0.06 (-0.03 - 0.14) | <0.001 |
| Triglyceride | 0.21 (0.13 - 0.30) | <0.001 |

**Additional File 1: Figures Legends**

**FigS1-** [Participants flowchart]

**FigS2-** [Study design and timeline of data collection]

**FigS3-** [Flowchart of SNP quality control (QC) steps and SNP retention]

**FigS4-** [Distribution of polygenic score for BMI]

**FigS5-** [Non-linear association between childhood UPF consumption and adulthood adiposity in 10 imputed data]
